# Age determination of vessel wall hematoma in spontaneous cervical artery dissection: A multi-sequence 3T Cardiovascular Magnetic resonance study

**DOI:** 10.1186/1532-429X-13-76

**Published:** 2011-11-28

**Authors:** Maximilian Habs, Thomas Pfefferkorn, Clemens C Cyran, Jochen Grimm, Axel Rominger, Marcus Hacker, Christian Opherk, Maximilian F Reiser, Konstantin Nikolaou, Tobias Saam

**Affiliations:** 1 Dept. of Clinical Radiology, University of Munich, Grosshadern Campus, Munich, Germany; 2 Dept. of Neurology, University of Munich, Grosshadern Campus, Munich, Germany; 3 Department of Nuclear Medicine, University of Munich, Grosshadern Campus, Munich, Germany

**Keywords:** CMR, internal carotid artery dissection, vertebral artery dissection, hematoma, stroke

## Abstract

**Background:**

Previously proposed classifications for carotid plaque and cerebral parenchymal hemorrhages are used to estimate the age of hematoma according to its signal intensities on T1w and T2w MR images. Using these classifications, we systematically investigated the value of cardiovascular magnetic resonance (CMR) in determining the age of vessel wall hematoma (VWH) in patients with spontaneous cervical artery dissection (sCAD).

**Methods:**

35 consecutive patients (mean age 43.6 ± 9.8 years) with sCAD received a cervical multi-sequence 3T CMR with fat-saturated black-blood T1w-, T2w- and TOF images. Age of sCAD was defined as time between onset of symptoms (stroke, TIA or Horner's syndrome) and the CMR scan. VWH were categorized into hyperacute, acute, early subacute, late subacute and chronic based on their signal intensities on T1w- and T2w images.

**Results:**

The mean age of sCAD was 2.0, 5.8, 15.7 and 58.7 days in patients with acute, early subacute, late subacute and chronic VWH as classified by CMR (p < 0.001 for trend). Agreement was moderate between VWH types in our study and the previously proposed time scheme of signal evolution for cerebral hemorrhage, Cohen's kappa 0.43 (p < 0.001). There was a strong agreement of CMR VWH classification compared to the time scheme which was proposed for carotid intraplaque hematomas with Cohen's kappa of 0.74 (p < 0.001).

**Conclusions:**

Signal intensities of VWH in sCAD vary over time and multi-sequence CMR can help to determine the age of an arterial dissection. Furthermore, findings of this study suggest that the time course of carotid hematomas differs from that of cerebral hematomas.

## Background

Spontaneous cervical artery dissection (sCAD) is an increasingly recognized cause of ischemic stroke, particularly in younger patients [1]. Timely diagnosis is mandatory, as instant anticoagulation or antithrombotic therapy can help to prevent more serious complications [2]. Clinical symptoms are often nonspecific. Therefore diagnosis may be facilitated by dedicated imaging techniques. Several studies have shown that cardiovascular magnetic resonance (CMR) is ideally suited to establish the diagnosis of sCAD by identifying the vessel wall hematoma (VWH) using fat suppressed T1-weighted images [3-5]. Due to the nonspecific symptoms of the disease, diagnosis is often delayed and it is often not possible to determine the exact onset of the patients' symptoms. Thus, the exact age of the arterial dissection often remains unknown. However, this information is useful as it has been shown that half of recurrent strokes/transient ischemic attacks in sCAD patients occur within the first 2 weeks after the event [6] and the anti-coagulation and anti-thrombotic therapies can be life threatening.

VWH is hypothesized to results from an intimal tear, allowing the blood to enter into the vessel wall, or from a rupture of the vasa vasorum. Both may lead to lumen stenosis or occlusion [7]. It is well known, that MR signal intensities of brain hematomas and hematomas in carotid atherosclerotic plaques change over time, due to the stepwise degradation of hemoglobin [8-11]. Gomori et al categorized cerebral hemorrhages into hyperacute (<24 hours), acute (1-3 days), early subacute (>3 days), late subacute (>7 days) and chronic (>14 days) ^11 ^according to its signal intensities on T1- and T2-weighted images. In 1982, Lusby at al proposed a histological classification of carotid hematomas into fresh (< 1 week), recent (1-6 weeks) and old (> 6 weeks) [12]. Chu et al. [13] showed that high-resolution CMR at 1.5T is able to identify hemorrhages into carotid atherosclerotic plaques (intraplaque hemorrhage = IPH) with good correlation to histopathology and that CMR is able to differentiate between different stages of hemorrhage according to the signal intensities on T1w and T2w images. However, a recent *in vivo *CMR study with 40 transient ischemic attack/stroke patients with ipsilateral <70% carotid stenosis showed that in 11 out of 12 patients with intraplaque hemorrhage at baseline, the hemorrhage signal remained unchanged over a 1-year period [14], indicating that the time course of carotid intraplaque hematomas differs from that of cerebral hematomas.

The purpose of our study was to use the above mentioned classifications for carotid plaque and cerebral parenchymal hemorrhages to systematically investigate the value of CMR in determining the age of vessel wall hematoma (VWH) in patients with spontaneous cervical artery dissection (sCAD).

## Methods

### Patient Selection

A total of 35 patients were enrolled in this prospective, monocentric observational study, which had been approved by the local institutional ethics committee. Patients with a first manifestation of spontaneous cervical artery dissection were included in the study if the following inclusion criteria were fulfilled: 1) no contraindication for CMR and unequivocal CMR evidence (intramural hematoma) of cervical artery dissection and 2) written informed consent. The date of dissection was estimated from the first appearance of one or more of the following symptoms or signs: Horner's syndrome, transient ischemic attack or stroke. If patients had > than one dissection only the artery was used for further analysis which contributed to the patients' symptoms (index artery). Patients with nonspecific symptoms (e.g. neck pain), a history of a related trauma, a pre-existing diagnosis of arteritis/vasculitis (e. g. Takayasu arteritis) or an underlying disease clearly associated with cervical artery dissection (e. g. Marfan syndrome) were excluded.

### MR Imaging Protocol

**Figure 1:**
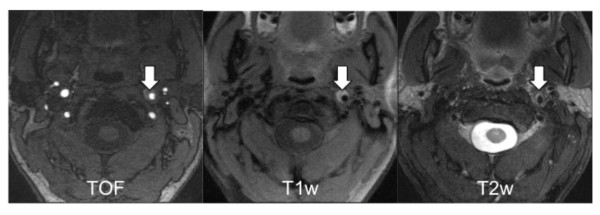
**Multi sequence CMR (TOF angiography, T1w- and T2w- images) of a 47-year old patient with spontaneous cervical artery dissection of the left ICA**. The patient presented with a left sided Horner's Syndrome. The VWH (arrows), bright in TOF and T1w images, originated from the proximal C1 segment of the ICA.

We used a multi-sequence CMR protocol ^15 ^without ECG gating or motion correction to image the cervical arteries with a 3.0 Tesla MR scanner (Magnetom Verio, Siemens Healthcare, Erlangen, Germany) using a 12-channel head coil and a dedicated four-channel surface coil (Machnet, Eelde, Netherlands). Each patient was scanned once with the same protocol which included a fat- and blood suppressed 2D-T1 Turbo Spin Echo (TSE) sequence [TR = 800 ms, TE = 12 ms], a fat- and blood suppressed 2D-T2 TSE sequence [TR = 3000 ms, TE = 65 ms] and a 3D-GRE time-of-flight angiography (TOF) [TR = 21 ms, TE = 3.96 ms]. Coverage was from the carotid bifurcation to the origin of the basilar artery. Best in plane resolution was 0.5 × 0.5 mm^2 ^with a slice thickness of 4 mm for T1- and T2- weighted images and 1 mm for TOF images with a field of view of 160*120 mm^2^. Number of slices was 20 - 30 for T1-, 24-36 for T2- and 104 for TOF images. Parallel-imaging with a PAT factor of 2 was used for all sequences using the generalized autocalibrating partially parallel acquisition algorithm (GRAPPA), resulting in a total scan time of 15-20 minutes, depending on the number of slices [15,16]. Figure 1 shows the applied sequences.

### Image Analysis

**Figure 2:**
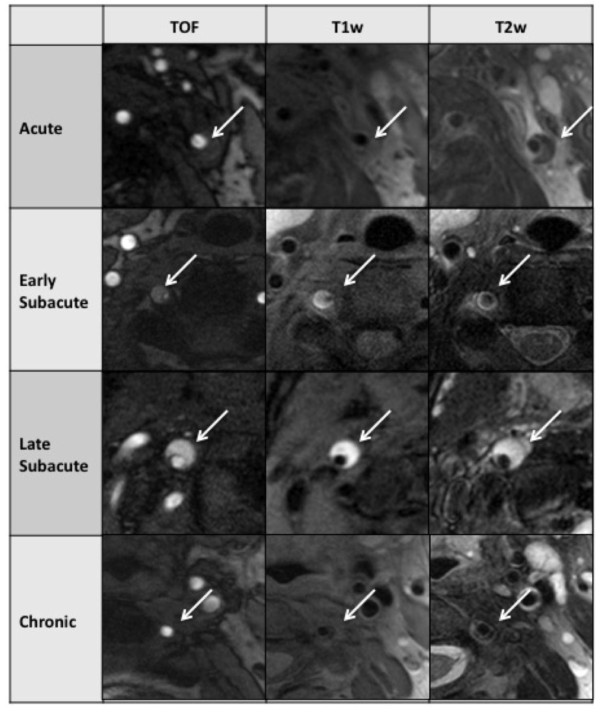
**Types of hemorrhage as determined by multi sequence CMR in different patients**. Line 1 shows an acute VWH of the left ICA, 2 days after onset of symptoms. Line 2 shows an early subacute VWH of the right VA with an imaging interval of 12 days. Line 3 shows a late subacute VWH of the right ICA after 17 days. Line 4 shows a chronic VWH of the left VA after 50 days.

Two radiologists with more than 5 years experience in black blood carotid artery imaging (T.S., K.N.) reviewed all cases and determined the affected vessels, the location of the VWH and its signal intensities. Both reviewers were blinded to clinical data. In case of any discrepant findings between the two readers, a final diagnosis was made in consensus. The age of hemorrhage within VWH was categorized on a 5-point scale into hyperacute, acute, early subacute, late subacute and chronic, based on the relative signal intensities of the hematoma on the T1w- and T2w- images compared to the normal vessel wall (in analogy to cerebral hemorrhage, Table 1). If vessel wall hematomas had mixed signal intensities, the hematoma was classified according to the oldest hemorrhage type present within the hematoma. If patients had more than one dissection, only the artery that contributed to the patient's symptoms was used for further analysis. Figure 2 shows examples for the different presentations of VWH signal intensities. Since a VWH can extend over several segments of an artery, its location was determined by the most proximal appearance. For the internal carotid artery (ICA) the beginning of the VWH was classified as either cervical/extracranial (C1 segment) or intracranial (C2 to C7 segment) [17]. The beginning of the VWH in the vertebral artery (VA) was described using the typical division of the VA into 4 segments (V1, V2, V3, V4) with V1 being the most proximal and V4 the intracranial part of the artery. The level of stenosis was evaluated according to the North American Symptomatic Carotid Endarterectomy Trial (NASCET) criteria on TOF images, i.e.comparing the measured lumen diameter at the site of dissection with the diameter at a more distal location with a normal lumen diameter.

**Table 1:** Relative signal intensities for different types of cerebral hemorrhage (adapted from Gomori et al [11])

Type of Hemorrhage	T1 w	T2 w	Histology
Hyperacute	-	↑	Intracellular oxyhemoglobin

Acute	↓ or -	↓	Intracellular deoxyhemoglobin

Early subacute	↑	↓	Intracellular methemoglobin

Late subacute	↑	↑	Extracellular methemoglobin

Chronic	↓	↓	Hemosiderin

All signal intensities relative to the normal carotid wall

↓ hypointense

↑ hyperintens

- isointens

### Classification Schemes of Cerebral and Carotid Hematomas

Gomori et al categorized cerebral hemorrhages into hyperacute (<24 hours), acute (1-3 days), early subacute (>3 days), late subacute (>7 days) and chronic (>14 days) [11] according to its signal intensities on T1- and T2-weighted images (see Table 1). In 1982, Lusby at al proposed a histological classification of carotid hematomas into fresh (< 1 week), recent (1-6 weeks) and old (> 6 weeks) based on the cellular features seen by elastochrome staining. Here fresh hemorrhage showed intact red blood cells, polymorph nuclear infiltrates and focal macrophage activity. Recent hemorrhage was characterized by hemorrhagic debris and macrophage engulfment of hemosiderin. Old hemorrhage was characterized by amorphous material surrounded by fibrous tissue [12].

### Statistical Analysis

Statistical analysis was carried out with SPSS version 16.0. We compared quantitative variables of groups using independent t-test. Differences of distribution regarding nominal variables in groups were analyzed with fisher's exact test. The level of agreement between hemorrhage classifications was measured using Cohen's kappa coefficient with values < 0 indicating no agreement and 0-0.20 as slight, 0.21-0.40 as fair, 0.41-0.60 as moderate, 0.61-0.80 as strong and 0.81-1.00 as almost perfect agreement. A p-value ≤ 0.05 (two-sided) was considered significant. Figures were generated using the software GraphPad Prism version 5.

## Results

### Patient Characteristics

43 patients with cervical artery dissection fulfilled the inclusion criteria. 8 patients were excluded due to traumatic dissection (n = 3), vasculitis (n = 2), nonspecific symptoms (n = 2), and fibromuscular dysplasia (n = 1). Table 2 gives an overview of the demographics, clinical presentation and cardiovascular risk factors. The mean age of all patients was 43.6 years, range 30-61 years. The mean interval between the onset of symptoms and the CMR scan was 22 ± 25 days. 26 out of 35 patients (74.3%) of our patients presented with a sensomotoric deficit as initial symptom and 9 patients (26.7%) had an isolated Horner's syndrome. 6 out of 35 patients (17.2%) had a Horner's syndrome *and *a sensomotoric deficit due to the sCAD. Of the 26 patients with a sensomotoric deficit, 16 were diagnosed with a stroke and 10 with a transient ischemic attack (TIA).

**Table 2:** Patient characteristics

	Study population (n = 35) Mean ± SD (range) or %
Age, y	43.6 ± 9.8 (30-61)

Male sex, %	60.0 (21/35)

BMI, kg/m^2^	24.2 ± 4.2 (19.7-41.7)

**MR Imaging**	

Age of the dissection	22.3 ± 24.9 (1-125)

Total occlusion, %	20.0 (7/35)

Stenosis, %	61.4 ± 33.9 (0-100)

*Internal Carotid Artery Dissection*	69 (24/35)

Extracranial C1 Segment, %	20 (5/24)

Intracranial C1 Segment, %	80 (19/24)

*Vertebral Artery Dissection*	31 (11/35)

V1 Segment, %	9 (1/11)

V2 Segment, %	55 (6/11)

V3 Segment, %	36 (4/11)

V4 Segment, %	0 (0/11)

*Type of Vessel Wall Hematoma*	

Acute, %	3 (1/35)

Early Subacute, %	17 (6/35)

Late Subacute, %	60 (21/35)

Chronic, %	20 (7/35)

**Symptoms**	

Stroke, %	45.7 (16/35)

Transient Ischemic Attack, %	28.6 (10/35)

Horner's Syndrome, %	42.9 (15/35)

**Cardiovascular Risk Factors**	

Current smoker, %	34.3 (12/35)

Former smoker, %	25.7 (9/35)

Hypertension, %	31.4 (11/35)

Hypercholesterolemia, %	25.7 (9/35)

Diabetes, %	0.0 (0/35)

Family history of cardiovascular events, %	17.1 (6/35)

### CMR Data

#### Vessels Affected and Degree of Stenosis

In 35 patients we observed a total of 47 sCAD. 18 patients (51%) presented with single ICA dissections, 7 patients (20%) presented with single VA dissections and 10 patients (29%) suffered from multiple artery dissections. Only the 35 index arteries which contributed to the patients' symptoms were used for further analysis. 24 index arteries were in the ICA and 11 in the VA. We detected 7 total occlusions through VWH or thrombus within 35 dissections (20%) and measured a mean level of stenosis in the non-occluded arteries of 61%. Although the percentage of stroke in single VA dissections tended to be higher than in single ICA dissections (71% vs. 40%), those findings were not statistically significant. Overall the occurrence of stroke was not associated with a significant higher level of stenosis (65% vs. 55%; p = 0.5). Patients with total occlusions of the affected artery tended to have a higher incidence of stroke (71% vs. 40%; p = 0.13).

VWH in ICA dissections was significantly more often seen in the intracranial portion (C2-C7 segments) than in the cervical portion (C1 segment) of the artery (80% vs. 20%; p < 0.05). The most common location of the VWH in VA dissections was the V2 segment (55%) and the V3 segment (36%). Only one VA dissection started in the V1 segment and no dissection originated from the V4 segment.

#### Age of Vessel Wall Hematoma in CMR

**Figure 3:**
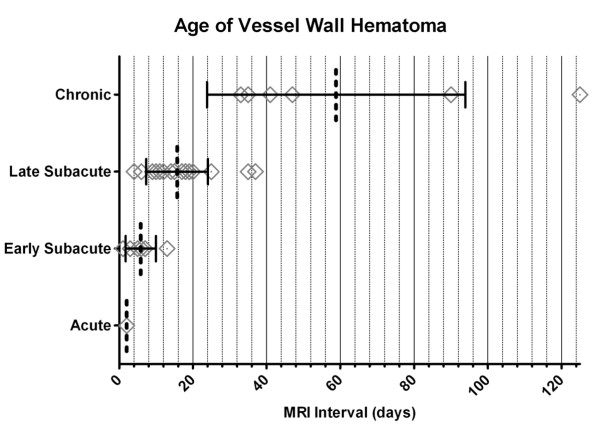
**Distribution of time intervals and type of hemorrhage as determined by CMR**. Dotted line indicates Mean. Whiskers indicate standard deviation of the Mean. Squares represent each patient.

**Figure 4:**
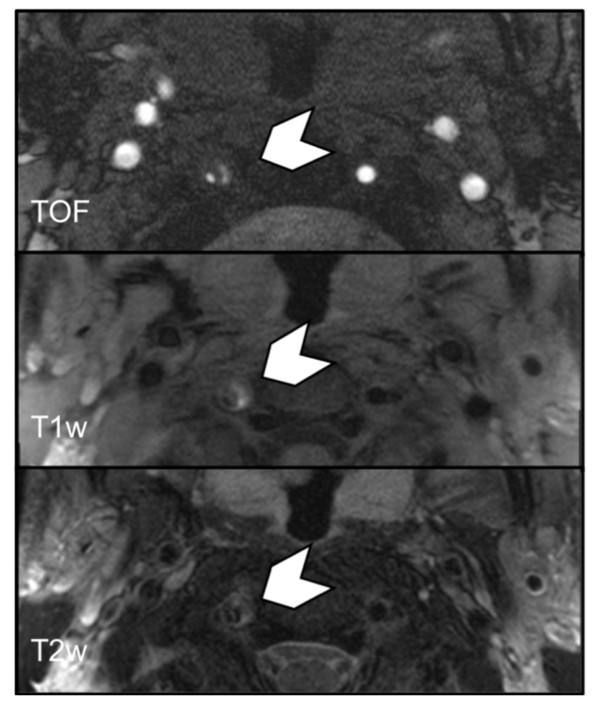
**Multi sequence CMR showing VWH (chevron) of the right VA in the V2 segment with mixed signal intensities in a 34-year-old patient with sCAD**. The hematoma presents with imaging features of acute, early subacute and late subacute hemorrhage. The VWH was classified according to the oldest hemorrhage type present (i.e. late subacute). CMR interval from onset of symptoms to scan was 9 days.

We observed no case of hyperacute hemorrhage. One patient who suffered a sCAD two days before, the CMR scan displayed imaging characteristics of an acute VWH. Six patients showed MR characteristics of early subacute hemorrhage with a mean imaging interval of 5.8 days, range 1-13 days. In 21 patients VWH was classified as late subacute hemorrhage according to CMR, with a mean imaging interval of 15.7 days, range 4-37 days. Seven patients showed MR characteristics of chronic VWH with a mean age of the dissection of 58.7 days, range 33-125 days. Mean age of sCAD was 2.0, 5.8, 15.7 and 58.7 days in patients with acute, early subacute, late subacute and chronic VWH (p < 0.001 for trend). There was only a moderate agreement between the chronological development of the MR signal changes seen in cerebral hemorrhage and the evolution of VWH in CMR with a Cohen's kappa of 0.43 (p < 0.001, see Table 3). However, when we compared our classification with the classification of carotid intraplaque hemorrhages which was proposed by Lusby et al in 1982 [12], there was strong agreement with a Cohen's kappa of 0.74 (p < 0.001, see Table 4). Figure 3 illustrates the presentation and typical signal characteristics of the different VWH types. 6 of the 35 index arteries (17%) had a VWH with mixed signal intensities and were classified according to the oldest component of hematoma (Figure 4).

**Table 3:** VWH age classified after criteria proposed by Gomori et al

	<1 Day	1-3 Days	4-7 Days	8-14 Days	>14 Days
**Observations (n = 35)**	**0**	**n = 2**	**n = 6**	**n = 10**	**n = 17**

Hyperacute (n = 0)	0	0	0	0	0

Acute (n = 1)	0	1	0	0	0

Early subacute (n = 6)	0	1	4	1	0

Late subacute (n = 21)	0	0	2	9	10

Chronic (n = 7)	0	0	0	0	7

**Table 4:** VWH age classified after criteria proposed by Lusby et al

	<1 Week	1-6 Weeks	>6 Weeks
**Observations (n = 35)**	**n = 8**	**n = 22**	**n = 5**

Fresh hemorrhage (n = 7)	6	1	0

Recent hemorrhage (n = 21)	2	19	0

Old hemorrhage (n = 7)	0	2	5

## Discussion

Our study showed that MR signal intensities of VWH are dependent on the age of the sCAD. More specifically, the age of the VWHs which were classified into acute, early subacute, late subacute and chronic hematomas according to its signal intensities on the T1w and T2w images differed, suggesting that *in vivo *CMR might be useful in determining the age of a sCAD. Given the fact that the risk of recurrent TIA or stroke is highest in the first two weeks after a sCAD [6] this information could be useful in patients with nonspecific initial symptoms, in whom the age of the sCAD is not known. From a clinical perspective, patients with more acute hematomas may therefore require a closer clinical observation than those with chronic hematomas. Furthermore, this information could be useful in cases where it is not clear whether the arterial dissection is spontaneous or traumatic. E.g., a patient with an arterial dissection and signs of an early subacute hemorrhage as determined by CMR, who suffered a car accident four weeks ago, will most likely have had a spontaneous dissection rather than a traumatic one. Finally, this multi-sequence MR protocol could give us new insights into the pathogenesis of the disease, especially in patients with multiple dissections, where the age of the VWH may be different for each individual affected vessel [18].

The MR signal changes observed in our study had only moderate agreement with the dynamics seen in cerebral hematomas (Cohen's kappa of 0.43; p < 0.001), suggesting that the time course of signal intensity changes varies in different body regions and organs. In contrast we found strong agreement with a previously proposed histology classification of carotid plaque hematomas [12] with a Cohen's kappa value of 0.74 (p < 0.001). However, in the study of carotid endarterectomy specimen of Lusby et al it is not clear how the age of the hematomas was determined and there is increasing evidence that MR signal characteristics of carotid intraplaque hematomas in atherosclerotic disease remain stable over a 1 year time period in most cases as has been suggested by Takaya et al [19] and Kwee et al [14]. These differences in the speed of hematoma decomposition might be explained by recurrent microbleeds into the atherosclerotic hematomas which are caused by leaky neovessels [20].

In the present study, 83% of the investigated VWH had a homogeneous appearance that could easily be staged due to their signal characteristics. The homogeneous appearance of the VWH supports the theory of a one-time event of sCAD in most cases. Only 17% of the VWH had mixed signal intensities, which could either indicate recurrent hemorrhages or a different speed of decomposition. All of these cases had a mix of acute, early and late subacute hematomas, which were then classified as late subacute hematomas (Figure 4).

Similar to previous studies, different segments of the VA and ICA have different propensities to develop a VWH [21-24]. More specifically, ICA dissections tend to occur significantly more often in the intracranial segment than in the cervical segment of the carotid artery (80% vs. 20%; p < 0.05). The most common location for VA dissections was the V2 (55%) and the V3 (36%) segment. Only one VA dissection started in the V1 segment and no dissection originated from the V4 segment. This is similar to findings of a study of 169 patients with VA dissection [22] who found incidences of 20%, 34%, 35% and 11% for the V1, V2, V3 and V4 segment, respectively.

About half the patients in our study (46%) developed a stroke. No significant relation was observed between the level of stenosis in dissected vessels and the occurrence of stroke (70% vs. 65%; p = 0.7). This supports the theory of Benninger et al [25] that a thrombembolic stroke-mechanism is probably more likely than hemodynamic effects in cervical artery dissection patients.

## Limitations

Although a CMR classification of VWH in sCAD patients seems feasible, our study has several limitations: since diagnostic workup for a patient with a fresh manifestation of cervical artery dissection takes time, we were not able to evaluate the hyperacute stage of hemorrhage and only one patient with the signal characteristics of acute hemorrhage was acquired. In general a longitudinal, rather than a cross-sectional study design would be more conclusive. Repeated scans of individual patients with cervical artery dissection within short intervals of time are necessary for a better understanding of the time course of the signals changes. Furthermore it can be difficult to determine the exact time between the occurrence of sCAD and the CMR scan. Therefore we included only patients with symptoms, such as TIA, stroke or Horner's syndrome, which could be clearly attributed to the sCAD. However, we cannot exclude that some patients might initially have had an asymptomatic sCAD and a recurrent episode of VWH a couple of days later causing the symptoms. However, the majority of our patients had homogeneous VWHs which could be clearly categorized into acute, early subacute, late subacute or chronic. Only 17% of all VWHs had mixed signal intensities, suggesting that either the speed of decomposition differed at different segments of the arteries or that these patients had a recurrence of VWH.

## Conclusions

Magnetic resonance imaging signal intensities of VWH in sCAD vary over time and multi-sequence CMR can help to determine the age of an arterial dissection. Furthermore, findings of this study suggest that the signal intensity change of carotid hematomas differs from that of cerebral hematomas, suggesting a different time course of decomposition. VWH classification by CMR in patients with sCAD could be useful to improve our understanding of the pathophysiology of the disease, by helping to identify potential causes of the disease, e.g. traumatic versus spontaneous dissections. It could also help to improve risk stratification of recurrent TIA/stroke especially in patients with nonspecific initial symptoms, where the age of the sCAD is not exactly known.

## Competing interests

The authors declare that they have no competing interests.

## Authors' contributions

MH, TP, CCC, JG, AR, MAH, CO, KN, MFR and TS were involved in the study concept/study. MH, TP and TS were involved in data analysis/interpretation. MH, TP, CCC, JG, AR, MAH, CO, KN, MFR and TS were involved in manuscript preparation and editing. MH, TP, CCC, JG, AR, MAH, CO, KN, MFR and TS gave final approval of the submitted manuscript.
